# Citizen Science for Water Quality Monitoring: Analyzing Key Parameters, Success Factors, and Research Gaps for Aquaculture

**DOI:** 10.1007/s00267-025-02261-8

**Published:** 2025-08-16

**Authors:** Walidatush Sholihah, Mohammad Gharesifard

**Affiliations:** 1 Faculty of Science and Engineering, University of Groningen, Groningen, The Netherlands; 2 Vocational School, IPB University, Bogor, West Java Indonesia

**Keywords:** Citizen science, Water quality, Aquaculture, Internet of things

## Abstract

Citizen science is often used as an approach for participatory water quality monitoring; however, its application in aquaculture remains limited. This study evaluates the current state of citizen science in monitoring water quality, with a particular focus on its implications for aquaculture practices. A systematic literature review was conducted using the Scopus and Web of Science databases to explore (1) common approaches for setting up and running such projects, (2) frequently used data collection tools and techniques, and (3) key water quality parameters. An in-depth review of 51 publications shows that citizen science studies are geographically concentrated in North America and Europe, with fewer studies in other regions. Most citizen science projects follow contributory models with a top-down approach, where scientists design research and citizens contribute data. Among the monitored parameters, chemical indicators, such as pH, are the most commonly monitored due to their significance in aquatic ecosystems and the availability of affordable test kits. These kits are widely used due to their ease of use and accessibility. However, the adoption of Internet of Things (IoT) technology remains limited, despite its potential to improve real-time monitoring and data accuracy. IoT-based systems, such as sensor boxes, can enhance citizen science by enabling automated data collection and expanding monitoring capabilities. Strengthening the integration of real-time monitoring technologies and broadening the range of monitored parameters could enhance the role of citizen science in aquaculture, supporting more effective and sustainable water quality management.

## Introduction

Aquaculture, particularly the cultivation of ornamental fish, requires optimal water quality to support fish health and their performance. Water quality affects fish health and performance, particularly their immunity, growth rate, and overall well-being (Mramba and Kahindi 2023; Nieman et al. 2020). Water quality, among other factors, influences fish development, especially during the juvenile and adult phases of their life cycles. Poor water quality can lead to various health issues, such as increased mortality and reduced aquaculture productivity (Wanja et al. 2020). Therefore, maintaining good water quality is essential for sustaining ornamental-fish farming.

Water quality is determined by a combination of physical, chemical, and biological parameters (Chidiac et al. 2023). The physical parameters include temperature, water clarity (color and turbidity), total dissolved solids (TDS), and total suspended solids (TSS) (Singh and Yadav 2022). Chemical parameters include ammonia, nitrite, nitrate, pH, and dissolved oxygen (Omer 2019). Biological parameters, including algae, bacteria, chlorophyll, and macroinvertebrates, provide insights into the ecological health of aquatic systems (Cuffney et al. 2014). Elevated concentrations of physical, chemical, or biological parameters can adversely affect water quality (Daru et al. 2024). Given the significance of these parameters, continuous monitoring is essential to maintain optimal water conditions and enhance the productivity of aquaculture.

Despite its importance, water quality monitoring in aquaculture faces significant challenges. Traditionally, it involves periodic sampling at specific locations, followed by laboratory analysis or the use of field-based equipment. Although these methods provide valuable data, they have some limitations. They are often expensive (Essamlali et al. 2024), cover only limited geographic areas (Bangira et al. 2024), rely on infrequent data collection (Farouk et al. 2023), and depend on specialized expertise (Amador-Castro et al. 2024). While fish farmers measure the water quality on their farms (Chen et al. 2022), another key limitation is the lack of knowledge exchange in the monitoring efforts across fish farms.

To address these limitations, citizen science has emerged as an innovative approach. Citizen science involves public participation in scientific research, enabling local communities to contribute to data collection and environmental monitoring (Fraisl et al. 2022). This approach has gained increasing attention as a way to complement traditional water quality monitoring, particularly in remote or underserved areas where expert-driven monitoring is limited.

Citizen science offers several advantages. Citizen science helps bridge data gaps by involving a wider network of participants who can conduct frequent and localized monitoring (Bonney 1996). It reduces costs by leveraging volunteer efforts rather than relying solely on professional resources (Poetz and Sauermann 2024). It also enhances environmental education and awareness, empowering community members to better understand water quality issues and take proactive measures (Larraz et al. 2024). Furthermore, citizen science strengthens collaboration between researchers and communities, allowing for mutual knowledge exchange (Starkey et al. 2024). This mutual learning process fosters collaboration, wherein researchers gain insights from local knowledge, and communities develop a deeper understanding of scientific approaches. The scalability of this method allows for simultaneous data collection across large geographic areas, producing comprehensive datasets that are essential for regional and global water quality assessments. Given these advantages, citizen science represents a powerful tool for enhancing water quality monitoring and ensuring broader societal engagement in the conservation of the environment.

Citizen science initiatives have been widely applied in water quality monitoring, utilizing various methods to assess the chemical, physical, and biological parameters. For chemical monitoring, citizen scientists often collect water samples and test for key indicators, such as pH, ammonia, nitrate, and dissolved oxygen, using test kits or portable devices (Metcalfe et al. 2022). Similarly, physical parameters, including temperature and turbidity, were measured using thermometers and turbidity tubes, respectively (Ho et al. 2020).

Biological monitoring methods are common in citizen science projects. For instance, the presence of algae is often recorded through visual documentation or photography (Kelly et al. 2016), whereas bacterial contamination is assessed by collecting samples for laboratory analysis (Roegner et al. 2017). Additionally, macroinvertebrate sampling is widely used to indicate ecosystem health using nets and other tools (Krabbenhoft and Kashian 2020; Peeters et al. 2022; Taylor et al. 2022). These diverse methodologies demonstrate the flexibility of citizen science in addressing various aspects of water quality.

Despite its success in environmental monitoring, citizen science remains underutilized in aquaculture. As parameters such as pH, dissolved oxygen, and turbidity significantly impact fish health and growth (Tumwesigye et al. 2022), involving fish farmers and local communities in monitoring efforts could provide valuable insights into aquaculture systems. Citizen science has the potential to support fish farmers in actively maintaining water quality. While its application in aquaculture remains limited, a notable example is provided by Elliott et al. (2019), who describe an integrated citizen science approach to monitoring fisheries and environmental data in the Cambodian Mekong. This early example highlights how participatory methods can be applied in aquaculture contexts, although such practices are not yet widespread. By using simple test kits or portable devices, fish farmers can conduct frequent assessments and take early preventive action when issues arise. This localized and participatory approach could benefit aquaculture practitioners by supporting the integration of scientific monitoring with traditional fish-farming practices.

Recent advancements in IoT technology, which refer to the connectivity of sensors and tools that can collect, transmit, and analyse data through networks (Arshi and Chaudhary 2024), have further expanded opportunities for citizen science in water quality monitoring. IoT-based water quality monitoring systems use sensors to measure water quality parameters. These devices can be directly installed in aquaculture systems, enabling continuous automated monitoring without manual intervention. Compared with traditional methods, IoT technology offers several advantages. This allows real-time data collection (Forhad et al. 2024), enabling fish farmers to detect and respond to water quality fluctuations immediately. It also reduces the costs and labor associated with manual sampling (Miller et al. 2023). Moreover, IoT devices facilitate automatic data storage and cloud-based analyses. This will provide stakeholders with long-term insights into the water quality trends. While IoT-based monitoring offers automated and real-time data collection, citizen science adds contextual insights, local knowledge, and community engagement that technology alone cannot provide. By combining both, aquaculture practitioners can achieve not only precise water-quality monitoring but also enhanced participation and sustainability.

Although citizen science and IoT-based water quality monitoring have been individually explored, their integration into aquaculture remains limited, leaving several research gaps. Most citizen science initiatives focus on general freshwater or marine ecosystems, with limited attention to fish farming systems. Likewise, while IoT devices are increasingly used for automated monitoring, their integration into community-driven, participatory models is still underdeveloped. There is limited understanding of how different citizen science models, whether contributory, collaborative, or co-created, affect the quality and usability of aquaculture data. Additionally, while various monitoring methods exist, including test kits, IoT devices, and manual sampling, comparative analyses of their effectiveness, accessibility, and suitability for aquaculture settings are still lacking. Furthermore, there is a lack of studies exploring the specific tools and techniques commonly used by citizen scientists for water quality data collection, particularly in aquaculture. Similarly, although water quality parameters such as pH, dissolved oxygen, and nutrient levels are widely monitored, there is still a need to identify which parameters are most relevant and practical for community-led monitoring efforts in aquaculture. To bridge these gaps, future research should explore the integration of citizen science and IoT in aquaculture monitoring, and assess their long-term potential to support sustainable, community-driven fish farming practices.

## Methods

This study followed the PRISMA framework (Page et al. 2021) to ensure a transparent and systematic process for identifying, screening and selecting studies relevant on citizen science in water quality monitoring, with a focus on aquaculture applications.

### Research questions

This review was guided by the following research questions:

**RQ1**: What are the **common approaches** to **setting up** and **running** citizen science projects for water quality monitoring in aquaculture?

**RQ2**: What are the most common **methods** used **for water quality monitoring** in aquaculture-related citizen science projects?

**RQ3**: What **water quality parameters** are commonly monitored in aquaculture-related citizen science projects?

### Literature Search

A comprehensive search was conducted on June 4, 2024, using the Scopus and Web of Science (WoS) databases. Keywords were structured around three main concepts: citizen science, water quality monitoring, and aquaculture. These concepts are combined using Boolean operators (“AND”, “OR”) and wildcards (*). The full keyword strategy is detailed in Table 1. Based on these rules, the following search string was constructed to identify relevant studies at the intersection of citizen science, water quality monitoring, and aquaculture: The keyword used as follows:

**Table 1:** Keywords used in the literature search

Aspect 1: The concept of citizen science	Aspect 2: Concept of water quality	Aspect 3: Concept of Aquaculture
Citizen science	water quality	Fish*
participatory research	water health	Aqua*
citizen observatory	water environment	Pisciculture
community-based monitoring	freshwater	Hatcheries
community-based sensing	pH	
community-based observation	Salinity	
participatory monitoring	Ammonia	
participatory sensing	Nitrate	
participatory observation	Nitrite	
collaborative monitoring	Turbidity	
collaborative sensing	acidity	
collaborative observation		
volunteer monitoring		
volunteer sensing		
volunteer observation		
citizen-based monitoring		
citizen-based sensing		
citizen-based observation		
public participation in science		
public participation in research		
public participation in scientific research		
participatory research		
participatory science		
science shop		

(“citizen science” OR “participatory research” OR “citizen observatory” OR “community-based monitoring” OR “community-based sensing” OR “community-based observation” OR “participatory monitoring” OR “participatory sensing” OR “participatory observation” OR “collaborative monitoring” OR “collaborative sensing” OR “collaborative observation” OR “volunteer monitoring” OR “volunteer sensing” OR “volunteer observation” OR “citizen-based monitoring” OR “citizen-based sensing” OR “citizen-based observation” OR “public participation in science” OR “public participation in research” OR “public participation in scientific research” OR “participatory research” OR “participatory science” OR “science shop”) AND (“water health” OR “water environment” OR “water quality” OR freshwater OR pH OR salinity OR ammonia OR nitrate OR nitrite OR turbidity OR acidity) AND (fish* OR aqua* OR pisciculture OR hatcheries).

The initial database search yielded 288 records. After removing duplicates using Mendeley reference manager, 230 unique studies remained. These studies were then screened based on the inclusion and exclusion criteria described in the following section.

### Inclusion and Exclusion Criteria

Establishing clear inclusion and exclusion criteria is essential for ensuring the quality and relevance of a systematic literature review (Meline 2006). The study selection followed a three-stage process: initiation, title and abstract screening, and full-text review. This procedure is adapted from Indriasari et al. (2024). Criteria focused on relevance to citizen science and water quality monitoring, with a particular interest in potential applications in aquaculture.

During the initiation stage, only English-language publications matching the keywords were retained. In the title and abstract screening, studies needed to mention at least one of the three key concepts: citizen science, water quality monitoring, or aquaculture. For full-text review, studies were included if they addressed citizen science in the context of water quality monitoring. While aquaculture was a desired focus, it was not mandatory, since only one study included all three concepts. Furthermore, simulation-only studies without field data were excluded.

At the end of this process, 51 studies were included for in-depth review. Table 2 summarizes the inclusion and exclusion criteria across each stage.

**Table 2:** Inclusion and exclusion criteria

Stages		Inclusion criteria	Exclusion criteria
1	Initiation	• According to the search keyword	• Language other than English
		• English	
2	Title and Abstract Selection	• Citizen science	• Not a citizen science project
		• Water quality monitoring	• Not discussed water quality monitoring
		• Aquaculture	
3	Full-Text Selection	• Citizen science project	• Does not discuss citizen science projects in water quality monitoring
		• Water quality monitoring	
		• Aquaculture	• Simulation-based studies without field data collection

### Data Extraction

**Fig. 1:**
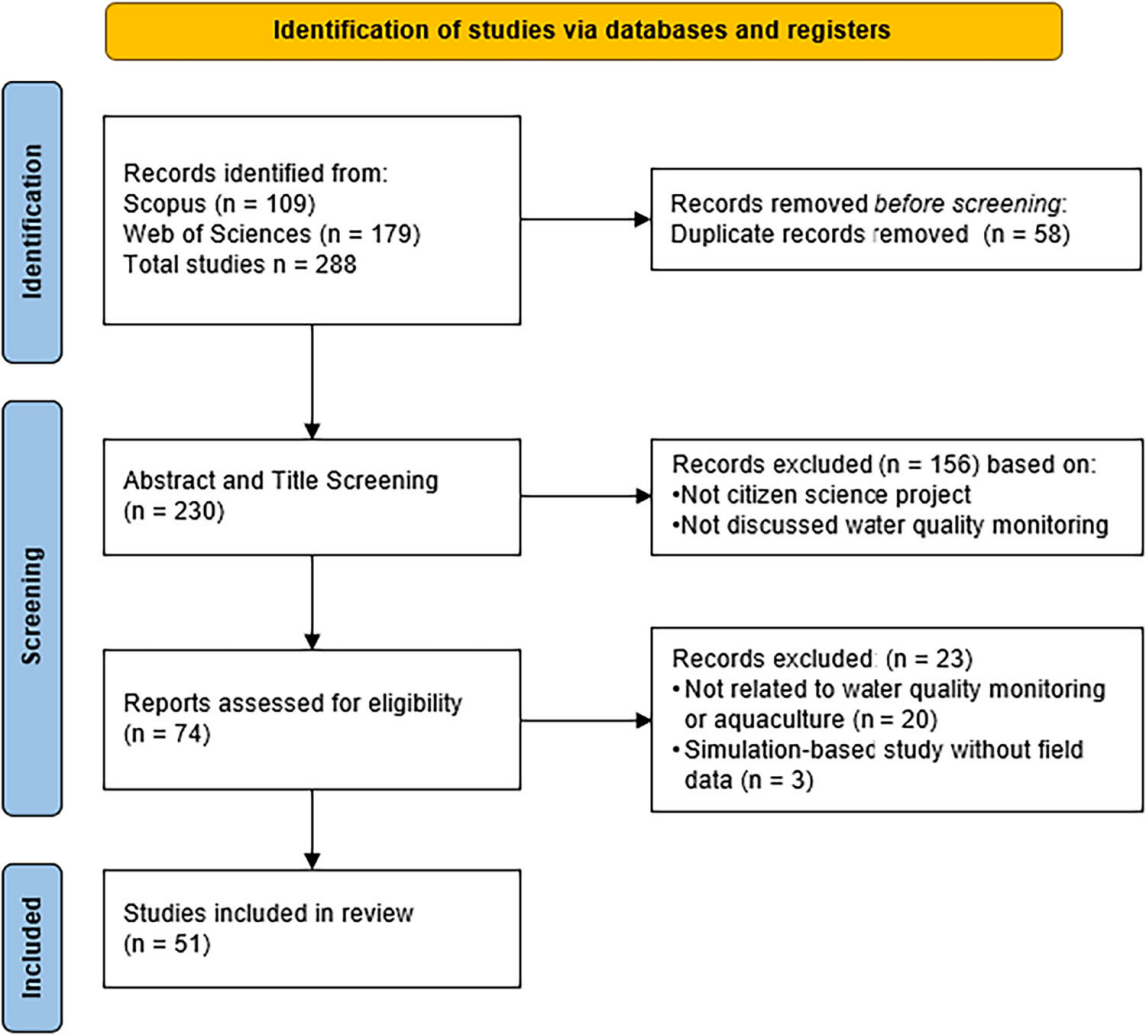
PRISMA diagram (inspired by Page et al. 2021)

The PRISMA Diagram (Fig. 1) illustrates the step-by-step selection process. A total of 51 studies met the inclusion criteria and were selected for in-depth analysis. Data were extracted on key aspects such as water quality parameters, measurement tools, geographic location, sampling methods, and citizen science typologies.

The studies were categorized based on five key dimensions. These include the type of citizen science model (contributory, collaborative, or co-created), the project initiator (top-down or bottom-up), the water quality parameters (e.g., pH, dissolved oxygen, temperature), the measurement tools used (IoT, test kits, or other techniques), and the geographical location.

Although 51 studies met the criteria, only one explicitly addressed all three key components of this review: citizen science, water quality monitoring, and aquaculture. The remaining studies focused on broader citizen science applications in water quality monitoring, contributing to a broader understanding of participatory monitoring approaches that may be applied within aquaculture systems.

## Results

### Common Typologies of Citizen Science Projects in Water Quality Monitoring

Citizen science projects may involve participants in different steps of scientific research, such as research design, data collection, analysis, and dissemination. However, the level of citizen involvement varies depending on the approach used. Shirk et al. (2012) divided citizen science projects into five models based on the degree of participation. **Contractual projects** involve communities commissioning professional researchers to conduct specific scientific investigations and report their findings. In, **Contributory projects**, scientists design the study, while public participants primarily contribute data. **Collaborative projects** go a step further, allowing the public to not only provide data but also e.g., help refine the project design, analyse results, and/or disseminate findings. **Co-created projects** are developed jointly by scientists and the public, with participants actively engaged in most or all aspects of the research. Lastly, **collegial contributions** involve independent research by non-credentialed individuals, with varying levels of recognition by institutional science. Each model differs in terms of citizen involvement, which influences data quality, project outcomes, and engagement levels.

While all 51 studies discussed citizen science in the context of water quality monitoring, only 24 of them provided explicit names of the citizen science projects involved. These specific projects were used as a basis for deeper analysis regarding project typologies (e.g., contributory, collaborative, or co-created), goals, and implementation structures. The other 27, were focusing on research related to citizen science and water quality monitoring without mentioning a specific project. More than half of these projects were contributory. Examples of such contributory projects include the FreshWater Watch, Florida Lakewatch and Stream Health Evaluation Program. These projects rely on volunteers to collect water samples and report their observations on the water quality. The analysis, design, and implementation of the project remain in researchers’ hands.

Examples of collaborative citizen science projects were found in only a few studies to date. These projects allow citizens to participate in data collection, design, data analysis, and interpretation. This approach encourages collaboration between researchers and volunteers. For example, SmartLagoon, IMALIRIJIIT, and the Cave Pearl Project involve partnerships in which citizens contribute data and participate in discussions on the results and potential actions.

Only one project, Gems of Water, was classified as a co-created citizen science project. In the context of this project, led by the JRC Water Laboratory and funded by the European Union, citizens help determine research questions, collect water samples, and analyse the results. They receive kits and laboratory tools to support their water quality monitoring work.

Citizen science projects can be categorized as top-down or bottom-up approaches based on who initiates the research (Ciravegna et al. 2013; Gharesifard et al. 2017). Some projects follow a top-down approach, where scientists design the study, define the research questions, and engage citizens primarily to assist with data collection. This model was the most common, with 19 of the 24 projects falling into this category. In these projects, actors such as research centers, environmental organizations, and government agencies lead initiatives. For example, the OPAL water survey in the UK was a nationwide citizen science project that engaged the public in monitoring water quality by testing pH, turbidity, and invertebrate diversity to assess the health of ecosystems. Similarly, the Michigan Clean Water Corps (MiCorps) in the United States is a long-standing program that trains volunteers to monitor lakes and streams.

In contrast, bottom-up citizen science projects are initiated by communities or citizen groups that independently monitor water quality to address local issues. This study categorized only five of the 24 projects as bottom-up initiatives. These projects empower local communities to define research priorities, select appropriate monitoring methods, and use data for local decision making. Examples of bottom-up projects include the Bellingen Riverwatch and Friends of Groundwater (FoG) projects. Bellingen Riverwatch is a community-led initiative in Australia, where local volunteers monitor river health to track water quality changes over time and provide data on environmental conservation. Similarly, Friends of Groundwater (FoG) is a grassroots initiative in which citizens work together to collect and analyse groundwater data. This project aims to raise public awareness among local communities and policymakers about groundwater issues and influence local water management policies. A full list of the 24 named projects, including their typologies and initiators, is provided in Table 3.

**Table 3:** Citizen science projects’ name, initiation, and typology

No	Project Name	Project initiation	Typology
1	Brooklyn Atlantis	Top-down	Contributory
2	Citclops	Top-down	Contributory
3	Freshwater Watch	Top-down	Contributory
4	OPAL water survey	Top-down	Contributory
5	HSBC Bank Water Program	Bottom-up	Contributory
6	Michigan Clean Water Corps (MiCorps)	Top-down	Contributory
7	Paddle surfing for science	Top-down	Contributory
8	The Walleye Tracker	Top-down	Contributory
9	The Citizens Statewide Lake Assessment Program (CSLAP)	Top-down	Contributory
10	Madcrow	Top-down	Contributory
11	Waterdiertjes.nl	Top-down	Contributory
12	MiniSASS	Top-down	Contributory
13	Smartlagoon	Top-down	Collaborative
14	Bellingen Riverwatch	Bottom-up	Contributory
15	Aquascope	Top-down	Contributory
16	Tracking change	Top-down	Collaborative
17	The Gems of Water	Top-down	Co-created
18	Florida Lakewatch	Top-down	Contributory
19	Anglers’ Riverfly Monitoring Initiative (ARMI)	Top-down	Contributory
20	Stream Health Evaluation Program	Top-down	Contributory
21	Cave Pearl Project	Bottom-up	Collaborative
22	Friends of Groundwater (FoG)	Bottom-up	Contributory
23	Assabet River StreamWatch project	Top-down	Contributory
24	IMALIRIJIIT	Bottom-up	Collaborative

### The Most Frequently used Data Collection Tools and Techniques for Water Quality Monitoring in Citizen Science Projects

**Fig. 2:**
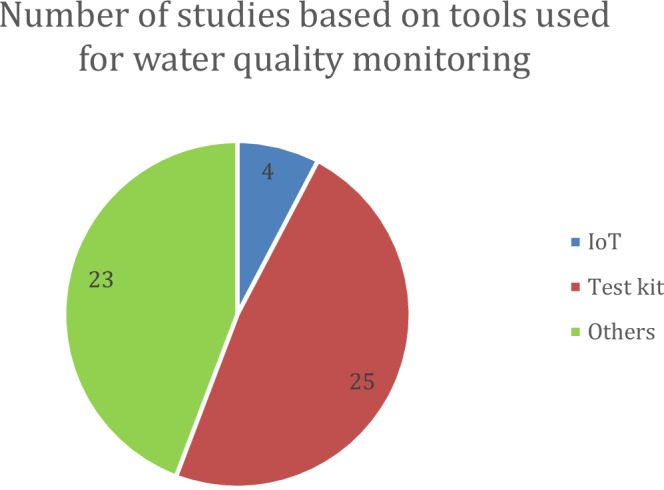
Number of studies based on tools used for water quality monitoring

Test kits emerged as the most commonly used data collection tools, found in 25 studies (Fig. 2). This could be related to the fact that test kits are the most accessible, affordable, and easy-to-use water quality monitoring methods used in citizen science projects (Aryal et al. 2024; Cacciatori et al. 2024; Cakmak et al. 2021). FreshWater Watch has developed test kits and a structured system that enables individuals, community groups, and researchers to monitor water quality and contribute to global water health assessments. The project is widely recognized and has been discussed in six studies. Although IoT technology has the potential to provide richer and more real-time data (Forhad et al. 2024; Jais et al. 2024; Langley et al. 2021), it has only been used in four studies. This may indicate barriers to accessing IoT technology in terms of cost, infrastructure, and technical skills required.

Several studies used more than one measurement method, indicating an attempt to obtain more complete and accurate data using a combination of methods (23 studies). In addition to the use of test kits and the IoT, other methods are used to measure water quality. For example, mobile surface vehicles (Laut et al. 2014) equipped with water quality sensors and cameras for aquatic environmental monitoring, Wi-Fi-enabled CTD (Kontoes et al. 2017) collect high-quality oceanographic data, paddle trawl devices (Camins et al. 2020) attached to a paddleboard investigate microplastic pollution in nearshore water, and a 3D printed mini Secchi disk (Menon et al. 2021) monitors changes in estuarine water quality. These diverse methods demonstrate the innovative approaches available for water quality monitoring, each with unique strengths tailored to specific environments and project requirements. They also demonstrate the potential for combining traditional tools with advanced technologies to achieve comprehensive and scalable monitoring solutions.

### Commonly Monitored Water Quality Parameters in Citizen Science Projects

As shown in Table 4, pH, dissolved oxygen, and turbidity were frequently measured water quality parameters. The widespread use of these parameter measurements in citizen science projects can be attributed to their cost-effectiveness, making them accessible to many participants. Additionally, pH testing is beneficial for large-scale surveys because of its simplicity and ease of use, enabling even non-professional volunteers to contribute to water quality monitoring efforts (Rose et al. 2016). The least measured parameter was nitrite content. This may be due to the difficulty and high cost of measuring these parameters.

**Table 4:** Distribution of measured parameters and water quality measurement techniques used across geographical locations in the studies analysed

Parameter category	Water quality parameter	# of studies	Water sampling method	Location
Chemical	pH	29	IoT, test kits, other method	North America, Europe, Asia, South America, Africa, Australia
	DO	19	IoT, test kits, other method	Australia, North America, Europe, Asia, Africa
	Nitrate	14	Test kits, other method	Australia, North America, Africa, Europe, Asia
	Nitrite	1	Test kits	North America
	Phosphorus	14	Test kits	Africa, North America
	Phosphate	9	Test kits	Australia, North America, Europe, Africa, Asia
	Salinity	6	IoT, other method	North America, Asia, Europe
Biological	Bacteria	5	Test kits, other method	South America, North America, Europe
	Algae	4	Other method	Europe
	Chlorophyll	11	IoT, test kits, other method	South America, North America, Africa
	Invertebrates	8	Test kits, other method	South America, North America, Europe, Asia, Africa
Physical	Turbidity	16	IoT, test kits, other method	Australia, Asia, North America, Europe, Africa
	TDS	4	Test kits, other device	Australia, North America, South America
	TSS	3	Test kits	North America, Africa
	Temperature	13	IoT, test kits, other method	Australia, Asia, North America, Europe, Africa
	water clarity	6	IoT, test kits, other method	Australia, North America, Europe, Asia

As shown in Table 4, the most commonly measured water quality parameter in citizen science projects was chemical, with pH appearing in more than half of the studies, followed by dissolved oxygen and nitrate. In contrast, nitrite was the least monitored parameter, with only one study including it. Biological parameters included chlorophyll (11 studies), invertebrates (8), algae (4), and bacteria (5). Physical parameters such as turbidity (16) and temperature (13) were also frequently recorded, while total dissolved solids and total suspended solids were reported in only 4 and 3 studies, respectively.

As shown in Table 5, North America accounted for over half of the reviewed studies (28 out of 51), making it the most represented region. Europe followed with 16 studies. South America, Africa, Asia, and Australia were each represented by fewer studies, ranging from 3 to 7 per region. The studies covered a range of countries, but the overall distribution indicates that most published citizen science projects on water quality monitoring originated from North America and Europe.

**Table 5:** The result of the geographical sampling location

Location of the project	Authors	# of Studies
North America	(Babich et al. 2021; Brewin et al. 2017; Cacciatori et al. 2024; Canfield et al. 2002; Casso-Hartmann et al. 2022; Field-Juma andRoberts-Lawler 2021; Fleming, 2003; Gérin-Lajoie et al. 2018; Hoyer and Canfield 2021; Jollymore et al. 2017; Jones et al. 2021; Kontoes et al. 2017; Kotovirta et al. 2015; Krabbenhoft and Kashian 2020; Laut et al. 2014; Lévesque et al. 2017; Louw and Sanford-Dolly 2024; Misstear et al. 2023; Nerbonne et al. 2008; Nieman et al. 2020; Parlee et al. 2021; Pérez-Belmont et al. 2019; Poisson et al. 2020; Roegner et al. 2017; Shupe, 2017; Taylor et al. 2022; Thornhill et al. 2018; Wang et al. 2020;)	28
South America	(Nabout et al. 2022; Taylor et al. 2022; Yevenes et al. 2022)	3
Europe	(Blanco-Gómez et al. 2023; Brewin et al. 2017; Busch et al. 2016; Camins et al. 2020; Diviacco et al. 2021; Kelly et al. 2016; Kotovirta et al. 2015; Marlowe et al. 2021; McGoff et al. 2017; Misstear et al. 2023; Moolna et al. 2020; Peeters et al. 2022; Rose et al. 2016; Taylor et al. 2022; Thornhill et al. 2018, Whitehead et al. 2024)	16
Asia	(Elliott et al. 2019; George et al. 2021; Ko et al. 2020; Kotovirta et al. 2015; Menon et al. 2021; Taylor et al. 2022; Yardi et al. 2019)	7
Africa	(Moshi, Kimirei, et al. 2022; Moshi, Shilla, et al. 2022; Ruppen et al. 2021; Taylor et al. 2022)	4
Australia	(Dickson et al. 2023; Korbel and Hose 2024; Thornhill et al. 2018)	3

Although IoT technology has great potential to improve monitoring accuracy and efficiency, its adoption is still minimal, as it was used in only four studies. In these projects, IoT devices, such as sensor boxes and portable monitoring units, were deployed to collect data on water quality parameters. Citizens participated by helping install and maintain the devices, retrieving data, or uploading readings to digital platforms. However, barriers to adopting this technology, such as cost, infrastructure, and technical skills, remain significant. These need to be addressed not only through research but also by increasing funding, implementing supportive policies, and encouraging the development of more affordable and accessible technologies. These combined efforts can help expand the use of IoT in citizen science projects.

## Discussion

This study systematically reviewed the application of citizen science in water quality monitoring, particularly its potential role in aquaculture. The results indicate that most citizen science projects for water quality monitoring follow a contributory model in which citizens participate primarily in data collection rather than research design or decision-making (Shirk et al. 2012). This approach is widely used because it enables engaging a large number of contributors, while allowing researchers to collect data using standardized methods. This aligns with the findings of Wadoux and McBratney (2023), who suggest that contributory models are often chosen because of their ability to engage many participants. In contrast, co-created approaches, in which communities are involved in defining research questions, designing protocols, and interpreting result, remain uncommon. The limited use of these more participatory models suggests an opportunity to explore how deeper community engagement could improve the relevance and sustainability of water quality monitoring, especially in aquaculture settings where local knowledge is essential. To fully harness citizen science in aquaculture, more inclusive models that allow for community input across multiple project stages may be necessary.

Most projects are initiated by scientists or governmental organizations following a top-down approach (Ciravegna et al. 2013; Gharesifard et al. 2017). Only a small number of studies in this review identified bottom-up citizen science projects in which communities independently initiate and manage monitoring efforts. Nevertheless, there are examples of successful community-led initiatives that highlight the value of a more demand-driven approach to participatory water quality monitoring (Gharesifard 2021). These findings support the broader objective of this paper, which is to identify not only the current state of citizen science practices but also areas where more participatory and context-specific models can be adopted for aquaculture applications.

We also found that chemical parameters were measured more often than biological parameters. This is because chemical monitoring often follows standardized protocols, making it easier to train volunteers and ensure consistency across the samples (Moshi, Shilla, et al. 2022). Projects such as the Riverfly Partnership in the UK have demonstrated the effectiveness of citizen science in monitoring both chemical and biological parameters. However, due to its relative simplicity, the focus has often remained on chemical monitoring (Moolna et al. 2020).

Among chemical parameters, pH was the most frequently measured, likely due to the widespread availability of affordable, easy-to-use tools that enable volunteers to collect data with minimal training. In contrast, parameters such as nitrite were rarely monitored, appearing in only one study, despite their ecological importance (Jones et al. 2021). The low adoption of nitrite monitoring may stem from its technical complexity, which requires careful sampling, controlled storage, and advanced laboratory techniques such as ion chromatography, barriers that limit feasibility for citizen science initiatives.

Monitoring biological parameters presents even greater challenges for non-experts. For example, studies in the Netherlands found that volunteers collected fewer animals and identified different species than professionals, highlighting the difficulty of obtaining reliable biological data without specialized training (Peeters et al. 2022). These findings suggest that further efforts are needed to improve volunteer training and methodological support, particularly for biological assessments.

This review also found that test kits are the most commonly used tools in citizen science projects for monitoring water quality. Their popularity can be attributed to their affordability, ease of use, and accessibility to non-experts (Aryal et al. 2024; Cacciatori et al. 2024; Cakmak et al. 2021). However, while test kits provide a simple and cost-effective solution for water quality monitoring, they have limitations in terms of continuous data collection and accuracy. To address these challenges, more advanced technologies such as IoT-based monitoring systems offer real-time data collection and improved measurement precision (Forhad et al. 2024; Jais et al. 2024). Despite this potential, the use of IoT technology for monitoring remains limited, and only a small fraction of the reviewed studies showcased IoT-based monitoring, possibly because of the costs, infrastructure limitations, and technical challenges. In addition, there is a perception that IoT systems reduce the need for direct human involvement, which may seem to conflict with the core idea of citizen science. However, this review suggests that IoT and citizen participation can be complementary. Citizens may contribute by installing, maintaining, and interpreting sensor data, especially in localized aquaculture settings where contextual knowledge is crucial.

Although IoT systems can be cost-effective in the long run, the initial setup costs, including purchasing sensors and developing the necessary infrastructure, can be high for many citizen science projects (Axiotidis et al. 2024). However, IoT technology remains a promising solution, particularly in aquaculture, where continuous and accurate water quality monitoring is essential. Integrating IoT technology into citizen science projects has great potential to improve data reliability (Jais et al. 2024), provide early warnings of water quality issues (Jais et al. 2024), and facilitate real-time decision-making by fish farmers (Shete et al. 2024). Importantly, citizen involvement in such projects does not have to be limited to data collection or device maintenance. In more participatory models, such as collaborative or co-created approaches, community members can also contribute to identifying local water quality issues, interpreting sensor data, and shaping context-specific monitoring strategies. These inclusive models may be especially valuable in aquaculture, where combining scientific tools with practitioner knowledge can lead to more sustainable and responsive management practices. Such integration reinforces the potential of citizen science as a practical and participatory solution to support sustainable aquaculture.

Although this review identified few published studies from regions such as Asia, Australia, and Africa, it is important to note that citizen science initiatives do exist in these areas. For example, projects like the Blue Map (China), MiniSASS (South Africa), and Atlas of Living Australia demonstrate active citizen engagement in environmental monitoring. However, many of these initiatives may prioritize local impact over academic publication and therefore are underrepresented in indexed literature databases.

A key limitation of this study is its reliance on the Scopus and Web of Science databases, which index only a subset of global academic publications. As a result, relevant citizen science initiatives may have been overlooked, particularly those published in non-indexed sources, such as government reports, local community projects documents, or regional repositories. Another possible reason for the underrepresentation of studies from certain regions is the variation in participant expertise, which can lead to inconsistencies in data quality and limit the potential for publication in peer-reviewed journals (Davis et al. 2023). Davis et al. (2023) also suggest that many citizen science initiatives prioritize community engagement and environmental education over academic research, resulting in fewer publication in scientific outlets.

Future systematic reviews could address these gaps by incorporating additional databases and gray literature to provide a more comprehensive picture of global citizen science efforts. As interest in participatory approaches grows, especially in sectors like aquaculture, understanding how to better document, support, and integrate citizen-driven efforts will be critical to advancing both science and sustainability.

## Conclusion

This study highlights the growing role of citizen science in water quality monitoring while emphasizing its limited application in aquaculture. Through a systematic literature review, we found that most citizen science projects follow a contributory model in which scientists design the research and citizens contribute data. Chemical indicators, particularly pH, are the most commonly monitored parameters due to their ecological significance and the accessibility of affordable test kits. However, despite its potential to enhance real-time data collection and accuracy, the adoption of IoT technology in citizen science remains minimal.

The findings suggest that expanding citizen science initiatives in aquaculture can improve sustainable water quality management by engaging local communities in data collection and decision-making. Additionally, integrating IoT-based monitoring systems, such as sensor boxes, can enhance data reliability and broaden the scope of the monitored parameters.

While this study provides valuable insights, it is limited by the geographical concentration of research in North America and Europe, with fewer studies conducted in other regions. Future research should explore citizen science applications in underrepresented areas and investigate alternative models that emphasize greater community involvement and leadership roles. Further studies on the integration of IoT and automated systems in citizen science could help optimize water quality monitoring for aquaculture. Strengthening these efforts could enhance the role of citizen science in promoting sustainable and effective aquaculture management.

## Data Availability

No datasets were generated or analysed during the current study.

## References

[CR1] Amador-Castro F, González-López ME, Lopez-Gonzalez G, Garcia-Gonzalez A, Díaz-Torres O, Carbajal-Espinosa O, Gradilla-Hernández MS (2024) Internet of things and citizen science as alternative water quality monitoring approaches and the importance of effective water quality communication. J Environ Manag 352:119959. 10.1016/j.jenvman.2023.119959. 10.1016/j.jenvman.2023.119959 38194871

[CR2] Arshi O, Chaudhary A (2024) Fundamental Concepts of IoT. In *Integration of Cloud Computing and IoT* (1st ed., p. 28). Chapman and Hall.

[CR3] Aryal P, Hefner CE, Martinez B, Brack E, Henry CS (2024) Citizen-based water quality monitoring: Field testing a user-friendly sensor for phosphate detection in global surface waters. Anal Chem 96(46):18369–18376. 10.1021/acs.analchem.4c02123. 39484865 10.1021/acs.analchem.4c02123

[CR4] Axiotidis C, Konstantopoulou E, Sklavos N (2024) A wireless sensor network IoT platform for consumption and quality monitoring of drinking water. Discov Appl Sci 7(1):15–35. 10.1007/s42452-024-06384-1.

[CR5] Babich R, Craig E, Muscat A, Disney J, Farrell A, Silka L, Jayasundara N (2021) Defining drinking water metal contaminant mixture risk by coupling zebrafish behavioral analysis with citizen science. Sci Rep 11(1). 10.1038/s41598-021-96244-4. 10.1038/s41598-021-96244-4 PMC8397788 34453073

[CR6] Bangira T, Matongera TN, Mabhaudhi T, Mutanga O (2024) Remote sensing-based water quality monitoring in African reservoirs, potential and limitations of sensors and algorithms: A systematic review. Phys Chem Earth, Parts A/B/C 134:103536. 10.1016/j.pce.2023.103536.

[CR7] Blanco-Gómez, P, Luis Jiménez-García, J, & Cecilia, JM (2023). Low-cost automated GPS, electrical conductivity and temperature sensing device (EC + T Track) and Android platform for water quality monitoring campaigns. HardwareX, 13. 10.5281/zenodo.6674534. 10.1016/j.ohx.2022.e00381 PMC9722445 36483327

[CR8] Bonney R (1996) Citizen science: A lab tradition. Living Bird 15(4):7–15.

[CR9] Brewin RJW, Hyder K, Andersson AJ, Billson O, Bresnahan PJ, Brewin TG, Cyronak T, Dall’Olmo G, Mora L de, Graham G, Jackson T, Raitsos DE (2017) Expanding aquatic observations through recreation. Front Marine Sci 4. 10.3389/fmars.2017.00351.

[CR10] Busch JA, Bardaji R, Ceccaroni L, Friedrichs A, Piera J, Simon C, Thijsse P, Wernand M, van derWoerd HJ, Zielinski O (2016) Citizen bio-optical observations from coast- and ocean and their compatibility with ocean colour satellite measurements. Remote Sensing, 8(11). 10.3390/rs8110879.

[CR11] Cacciatori C, Mariani G, Comero S, Marin D, Cabrera M, Bon-Tavarnese J, Gaggstatter J, Tavazzi S, Maffettone R, Myers J, Pettigrove V, Gawlik BM (2024) “The Gems of Water”: A co-created scientist-citizen approach for water quality monitoring. Front Water, 6. 10.3389/frwa.2024.1358959.

[CR12] Cakmak EK, Ugurlu A, Anbaroglu B (2021) Adopting citizen science approach for water quality monitoring in Uzungöl, Turkey. Environ Monit Assess 193(9):604. 10.1007/s10661-021-09395-2. 34448950 10.1007/s10661-021-09395-2

[CR13] Camins E, de Haan WP, Salvo VS, Canals M, Raffard A, Sanchez-Vidal A (2020) Paddle surfing for science on microplastic pollution. Sci Total Environ 709. 10.1016/j.scitotenv.2019.136178. 10.1016/j.scitotenv.2019.136178 31884295

[CR14] Canfield DE, Brown CD, Bachmann RW, Hoyer MV (2002) Volunteer lake monitoring: Testing the reliability of data collected by the Florida Lakewatch program. Lake Reserv Manag 18(1):1–9.

[CR15] Casso-Hartmann L, Rojas-Lamos P, McCourt K, Vélez-Torres I, Barba-Ho LE, Bolaños BW, Montes CL, Mosquera J, Vanegas D (2022) Water pollution and environmental policy in artisanal gold mining frontiers: The case of La Toma, Colombia. Sci Total Environ 852. 10.1016/j.scitotenv.2022.158417. 10.1016/j.scitotenv.2022.158417 36055504

[CR16] Chen C-H, Wu Y-C, Zhang J-X, Chen Y-H (2022) IoT-based fish farm water quality monitoring system. Sensors 22(17):6700. 10.3390/s22176700. 36081159 10.3390/s22176700 PMC9460614

[CR17] Chidiac S, Najjar PE, Ouaini N, Rayess YE, Azzi DE(2023) A comprehensive review of water quality indices (WQIs): History, models, attempts and perspectives Rev Environ Sci Biotechnol 22:349–395. 10.1007/s11157-023-09650-7 37234131 10.1007/s11157-023-09650-7 PMC10006569

[CR18] Ciravegna F, Huwald H, Lanfranchi V, Wehn de Montalvo U (2013) Citizen Observatories: The WeSenseIt vision. *Infrastructure for Spatial Information in the European Community*. INSPIRE 2013, Florence, Italy.

[CR19] Cuffney TF, Kennen JG, Waite IR (2014) Aquatic ecosystems as indicators of status and trends in water quality. In S. Ahuja (Ed.), *Comprehensive Water Quality and Purification* (pp. 122–156). Elsevier. 10.1016/B978-0-12-382182-9.00008-6.

[CR20] Daru AF, Susanto S, Adhiwibowo W (2024) Arowana cultivation water quality monitoring and prediction using autoregressive integrated moving average. Int J Reconfigurable Embedded Syst (IJRES) 13(3):665–673. 10.11591/ijres.v13.i3.pp665-673.

[CR21] Davis LS, Zhu L, Finkler W (2023) Citizen science: Is it good science? Sustainability, 15(5), Article 5. 10.3390/su15054577.

[CR22] Dickson A, Belmer N, Denshire A, Garland I, Lennox S, Ruming S, Lawler D, Wethered A (2023) Can citizen science inform science? Evaluating the results of the Bellingen Riverwatch citizen science program and a complimentary government monitoring program. Front Environ Sci 11. 10.3389/fenvs.2023.1237580.

[CR23] Diviacco P, Nadali A, Iurcev M, Carbajales R, Busato A, Pavan A, Burca M, Grio L, Nolich M, Molinaro A, Malfatti F (2021) MaDCrow, a citizen science infrastructure to monitor water quality in the Gulf of Trieste (North Adriatic Sea). Front Marine Sci 8. 10.3389/fmars.2021.619898.

[CR24] Elliott VL, Chheng P, Uy S, Holtgrieve GW (2019) Monitoring of tropical freshwater fish resources for sustainable use. J Fish Biol 94(6):1019–1025. 10.1111/jfb.13974. 30950505 10.1111/jfb.13974

[CR25] Essamlali I, Nhaila H, El Khaili M (2024) Advances in machine learning and IoT for water quality monitoring: A comprehensive review. Heliyon 10(6):e27920. 10.1016/j.heliyon.2024.e27920. 38533055 10.1016/j.heliyon.2024.e27920 PMC10963334

[CR26] Farouk MIHZ, Jamil Z, Abdul Latip MF (2023) Towards online surface water quality monitoring technology: A review. Environ Res 238:117147. 10.1016/j.envres.2023.117147. 37716398 10.1016/j.envres.2023.117147

[CR27] Field-Juma A, Roberts-Lawler N (2021) Using partnerships and community science to protect wild and scenic rivers in the Eastern United States. Sustainability (Switz) 13(4):1–22. 10.3390/su13042102.

[CR28] Fleming W (2003) Volunteer watershed health monitoring by local stakeholders: New mexico watershed watch. J Environ Educ 35(1):27–32. 10.1080/00958960309600592.

[CR29] Forhad HM, Uddin MdR, Chakrovorty RS, Ruhul AM, Faruk HM, Kamruzzaman S, Sharmin N, Jamal ASIM, Haque MdM-U, Morshed AM (2024) IoT based real-time water quality monitoring system in water treatment plants (WTPs). Heliyon 10(23):e40746. 10.1016/j.heliyon.2024.e40746. 39698090 10.1016/j.heliyon.2024.e40746 PMC11652906

[CR30] Fraisl D, Hager G, Bedessem B, Gold M, Hsing P-Y, Danielsen F, Hitchcock CB, Hulbert JM, Piera J, Spiers H, Thiel M, Haklay M (2022) Citizen science in environmental and ecological sciences. Nat Rev Methods Prim 2(1):1–20. 10.1038/s43586-022-00144-4.

[CR31] George G, Menon NN, Abdulaziz A, Brewin RJW, Pranav P, Gopalakrishnan A, Mini KG, Kuriakose S, Sathyendranath S, Platt T (2021) Citizen scientists contribute to real-time monitoring of lake water quality using 3D printed mini secchi disks. Front Water, 3. 10.3389/frwa.2021.662142.

[CR32] Gérin-Lajoie J, Herrmann TM, MacMillan GA, Hébert-Houle É, Monfette M, Rowell JA, Soucie TA, Snowball H, Townley E, Lévesque E, Amyot M, Franssen J, Dedieu JP (2018) IMALIRIJIIT: a community-based environmental monitoring program in the George River Watershed, Nunavik,Canada. Ecoscience 25(4):381–399. 10.1080/11956860.2018.1498226.

[CR33] Gharesifard M (2021) *Community-Based Monitoring Initiatives of Water and Environment: Evaluation of Establishment Dynamics and Results* (1st ed.). CRC Press. 10.1201/9781003131243.

[CR34] Gharesifard M, Wehn U, Van Der Zaag P (2017) Towards benchmarking citizen observatories: Features and functioning of online amateur weather networks. J Environ Manag 193:381–393. 10.1016/j.jenvman.2017.02.003 28249761

[CR35] Ho SY-F, Xu SJ, Lee FW-F (2020) Citizen science: An alternative way for water monitoring in Hong Kong. PLOS ONE. 10.1371/journal.pone.0238349. 10.1371/journal.pone.0238349 PMC7478504 32898181

[CR36] Hoyer MV, Canfield DE (2021) Volunteer-collected water quality data can be used for science and management. Lake Reserv Manag 37(3):235–245. 10.1080/10402381.2021.1876190.

[CR37] Indriasari S, Sensuse DI, Resti Y, Wurzinger M, Hidayat DS, Widodo B (2024) Requirements engineering of knowledge management system for smallholder dairy farmers. J Hum, Earth, Future 5(2):Article 2. 10.28991/HEF-2024-05-02-02.

[CR38] Jais NAM, Abdullah AF, Mohd Kassim MS, Abd Karim M, M, M A, Muhadi N (2024) Improved accuracy in IoT-Based water quality monitoring for aquaculture tanks using low-cost sensors: Asian seabass fish farming. Heliyon 10(8):e29022. 10.1016/j.heliyon.2024.e29022. 38655304 10.1016/j.heliyon.2024.e29022 PMC11035052

[CR39] Jollymore A, Haines MJ, Satterfield T, Johnson MS (2017) Citizen science for water quality monitoring: Data implications of citizen perspectives. J Environ Manag 200:456–467. 10.1016/j.jenvman.2017.05.083. 10.1016/j.jenvman.2017.05.083 28618317

[CR40] Jones EF, Frei RJ, Lee RM, Maxwell JD, Shoemaker R, Follett AP, Lawson GM, Malmfeldt M, Watts R, Aanderud ZT, Allred C, Asay AT, Buhman M, Burbidge H, Call A, Crandall T, Errigo I, Griffin NA, Hansen NC, … Abbott BW (2021) Citizen science reveals unexpected solute patterns in semiarid river networks. PLoS ONE, 16(8 August). 10.1371/journal.pone.0255411. 10.1371/journal.pone.0255411 PMC8376020 34411107

[CR41] Kelly MG, Krokowski J, Harding JPC (2016) RAPPER: A new method for rapid assessment of macroalgae as a complement to diatom-based assessments of ecological status. Sci Total Environ 568:536–545. 10.1016/j.scitotenv.2015.12.068. 26767621 10.1016/j.scitotenv.2015.12.068

[CR42] Ko NT, Suter P, Conallin J, Rutten M, Bogaard T (2020) Aquatic macroinvertebrate community changes downstream of the hydropower generating dams in Myanmar-potential negative impacts from increased power generation. Front Water 2. 10.3389/frwa.2020.573543.

[CR43] Kontoes CP, Flagg R, Gawarkiewicz G, Bahr F, Winsor P, Danielson SL (2017) Wi-Fi enabled CTDs in citizen science. OCEANS 2017 - Anchorage, 2017, 1–5.

[CR44] Korbel KL, Hose GC (2024) Monitoring Groundwater Health Using Citizen Scientists in Semi-Arid Regional Australia. Groundwater. 10.1111/gwat.13407. 10.1111/gwat.13407 38572675

[CR45] Kotovirta V, Toivanen T, Tergujeff R, Häme T, Molinier M (2015) Citizen science for earth observation: Applications in environmental monitoring and disaster response. Int Arch Photogramm, Remote Sens Spat Inf Sci - ISPRS Arch 40(7W3):1221–1226. 10.5194/isprsarchives-XL-7-W3-1221-2015.

[CR46] Krabbenhoft CA, Kashian DR (2020) Citizen science data are a reliable complement to quantitative ecological assessments in urban rivers. Ecological Indicators, 116. 10.1016/j.ecolind.2020.106476.

[CR47] Langley DJ, van Doorn J, Ng ICL, Stieglitz S, Lazovik A, Boonstra A (2021) The Internet of Everything: Smart things and their impact on business models. J Bus Res 122:853–863. 10.1016/j.jbusres.2019.12.035.

[CR48] Larraz B, Urquiaga R, Martínez A, Martín B (2024) Improving knowledge and awareness and contributing to policy making on river pressures through a citizen science approach: Tagus web viewer case (Spain). Water 16(15):Article 15. 10.3390/w16152214.

[CR49] Laut J, Henry E, Nov O, Porfiri M (2014) Development of a mechatronics-based citizen science platform for aquatic environmental monitoring. IEEE/ASME Trans Mechatron 19(5):1541–1551. 10.1109/TMECH.2013.2287705.

[CR50] Lévesque D, Cattaneo A, Deschamps G, Hudon C (2017) In the eye of the beholder: Assessing the water quality of shoreline parks around the Island of Montreal through citizen science. Sci Total Environ 579:978–988. 10.1016/j.scitotenv.2016.10.175. 27914646 10.1016/j.scitotenv.2016.10.175

[CR51] Louw M, Sanford-Dolly CW (2024) Learning to see, seeing to learn: Impacts of an online tool on volunteers’ observational practices during aquatic macroinvertebrate identification. Sci Educ 108(1):332–364. 10.1002/sce.21834.

[CR53] Marlowe, C, Hyder, K, Sayer, MDJ, & Kaiser, J (2021). Divers as citizen scientists: Response time, accuracy and precision of water temperature measurement using dive computers. Front Marine Sci 8. 10.3389/fmars.2021.617691.

[CR54] McGoff E, Dunn F, Cachazo LM, Williams P, Biggs J, Nicolet P, Ewald NC (2017) Finding clean water habitats in urban landscapes: Professional researcher vs citizen science approaches. Sci Total Environ 581–582:105–116. 10.1016/j.scitotenv.2016.11.215. 10.1016/j.scitotenv.2016.11.215 28069307

[CR55] Meline T (2006) Selecting studies for systemic review: Inclusion and exclusion criteria. Contemp Issues Commun Sci Disord 33(Spring):21–27. 10.1044/cicsd_33_S_21.

[CR56] Menon N, George G, Ranith R, Sajin V, Murali S, Abdulaziz A, Brewin RJW, Sathyendranath S (2021) Citizen science tools reveal changes in estuarine water quality following demolition of buildings. Remote Sensing, 13(9). 10.3390/rs13091683.

[CR57] Metcalfe AN, Kennedy TA, Mendez GA, Muehlbauer JD (2022) Applied citizen science in freshwater research. Wiley Interdisciplinary Rev Water, 9(2). 10.1002/wat2.1578.

[CR58] Miller M, Kisiel A, Cembrowska-Lech D, Durlik I, Miller T (2023) IoT in water quality monitoring—are we really here?. Sensors 23(2):960. 10.3390/s23020960. 36679757 10.3390/s23020960 PMC9864729

[CR59] Misstear B, Vargas CR, Lapworth D, Ouedraogo I, Podgorski J (2023) A global perspective on assessing groundwater quality. Hydrogeol J 31:11–14. 10.1007/s10040-022-02461-0/Published.

[CR60] Moolna A, Duddy M, Fitch B, White K (2020) Citizen science and aquatic macroinvertebrates: Public engagement for catchment-scale pollution vigilance. Ecoscience 27(4):303–317. 10.1080/11956860.2020.1812922.

[CR61] Moshi HA, Kimirei I, Shilla D, O’Reilly C, Wehrli B, Ehrenfels B, Loiselle S (2022) Citizen scientist monitoring accurately reveals nutrient pollution dynamics in Lake Tanganyika coastal waters. Environ Monitoring Assessment, 194(10). 10.1007/s10661-022-10354-8. 10.1007/s10661-022-10354-8 PMC9391239 35984535

[CR62] Moshi HA, Shilla DA, Kimirei IA, O’Reilly C, Clymans W, Bishop I, Loiselle SA (2022) Community monitoring of coliform pollution in Lake Tanganyika. PLoS ONE, 17(1 January). 10.1371/journal.pone.0262881. 10.1371/journal.pone.0262881 PMC8797266 35089939

[CR63] Mramba RP, Kahindi EJ (2023) Pond water quality and its relation to fish yield and disease occurrence in small-scale aquaculture in arid areas. Heliyon 9(6):e16753. 10.1016/j.heliyon.2023.e16753. 37274696 10.1016/j.heliyon.2023.e16753 PMC10238929

[CR64] Nabout JC, David ACM, Felipe JF, Machado KB, Carvalho L, da Cunha HF (2022) Can people detect the loss of water quality? A field experiment to evaluate the correlation between visual perception and water eutrophication degree. Acta Limnologica Brasiliensia, 34. 10.1590/S2179-975X2921.

[CR65] Nerbonne JF, Ward B, Ollila A, Williams M, Vondracek B (2008) Effect of sampling protocol and volunteer bias when sampling for macroinvertebrates. J North Am Benthol Soc 27(3):640–646. 10.1899/07-101.1.

[CR66] Nieman CL, Bruskotter JT, Braig EC, Gray SM (2020) You can’t just use gold: Elevated turbidity alters successful lure color for recreational Walleye fishing. J Gt Lakes Res 46(3):589–596. 10.1016/j.jglr.2020.03.002.

[CR67] Omer NH (2019) Water Quality Parameters. In *Water Quality—Science, Assessments and Policy*. IntechOpen. 10.5772/intechopen.89657.

[CR68] Page MJ, Moher D, Bossuyt PM, Boutron I, Hoffmann TC, Mulrow CD, Shamseer L, Tetzlaff JM, Akl EA, Brennan SE, Chou R, Glanville J, Grimshaw JM, Hróbjartsson A, Lalu MM, Li T, Loder EW, Mayo-Wilson E, McDonald S, … McKenzie JE (2021). *PRISMA 2020 explanation and elaboration: Updated guidance and exemplars for reporting systematic reviews*. 10.1136/bmj.n160. 10.1136/bmj.n160 PMC8005925 33781993

[CR69] Parlee B, Huntington H, Berkes F, Lantz T, Andrew L, Tsannie J, Reece C, Porter C, Nicholson V, Peter S, Simmons D, Michell H, Lepine M, Maclean B, Ahkimnachie K, King LJ, Napoleon A, Hogan J, Lam J, … Howlett T (2021) One-size does not fit all—A networked approach to community-based monitoring in large river basins. Sustainability (Switzerland), 13(13). 10.3390/su13137400.

[CR70] Peeters ETHM, Gerritsen AAM, Seelen LMS, Begheyn M, Rienks F, Teurlincx S (2022) Monitoring biological water quality by volunteers complements professional assessments. PLoS ONE, 17(2 February). 10.1371/journal.pone.0263899. 10.1371/journal.pone.0263899 PMC8880917 35213583

[CR71] Pérez-Belmont P, Alvarado J, Vázquez-Salvador N, Rodríguez E, Valiente E, Díaz J (2019) Water quality monitoring in the Xochimilco peri-urban wetland: Experiences engaging in citizen science. Freshw Sci 38(2):342–351. 10.1086/703395.

[CR72] Poetz MK, Sauermann H (2024) Citizen Science and Crowd Science. *Oxford University Press*. 10.1093/acrefore/9780190224851.013.385.

[CR73] Poisson AC, McCullough IM, Cheruvelil KS, Elliott KC, Latimore JA, Soranno PA (2020) Quantifying the contribution of citizen science to broad-scale ecological databases. Front Ecol Environ 18(1):19–26. 10.1002/fee.2128.

[CR74] Roegner A, Ochaeta G, Bocel E, Ogari Z, Pfotenhaeur B, Rejmankova E (2017) Employing CBPR to investigate function, utility, and longevity of household filters to improve potable water quality for indigenous peoples at Lake Atitlán, Guatemala: A pilot study with San Pedro de La Laguna. Energy, Ecol Environ 2(2):95–113. 10.1007/s40974-016-0045-4. 32280742 10.1007/s40974-016-0045-4 PMC7147507

[CR75] Rose NL, Turner SD, Goldsmith B, Gosling L, Davidson TA (2016) Quality control in public participation assessments of water quality: The OPAL Water Survey. BMC Ecol, 16. 10.1186/s12898-016-0063-2. 10.1186/s12898-016-0063-2 PMC4965718 27459958

[CR76] Ruppen D, Chituri OA, Meck ML, Pfenninger N, Wehrli B (2021). Community-based monitoring detects sources and risks of mining-related water pollution in Zimbabwe. Front Environ Sci 9. 10.3389/fenvs.2021.754540.

[CR77] Shete RP, Bongale AM, Dharrao D (2024) IoT-enabled effective real-time water quality monitoring method for aquaculture. MethodsX 13:102906. 10.1016/j.mex.2024.102906. 39263361 10.1016/j.mex.2024.102906 PMC11387385

[CR78] Shirk JL, Ballard HL, Wilderman CC, Phillips T, Wiggins A, Jordan R, McCallie E, Minarchek M, Lewenstein BV, Krasny ME, Bonney R (2012) Public participation in scientific research: A framework for deliberate design. Ecol Soc 17(2):art29. 10.5751/ES-04705-170229.

[CR79] Shupe SM (2017) High resolution stream water quality assessment in the Vancouver, British Columbia region: A citizen science study. Sci Total Environ 603–604:745–759. 10.1016/j.scitotenv.2017.02.195. 10.1016/j.scitotenv.2017.02.195 28411868

[CR80] Singh M, Yadav R (2022) Physico-chemical parameters for water quality check: A comprehensive review. Int J Res Anal Rev (IJRAR 9(4):287–293. 10.13140/RG.2.2.11070.25929

[CR81] Starkey E, Jones A, Ochoa-Rodriguez S, Mahajan S, Wei C-L, Chen P-C, Liu S-Y, Wang L-P, Walsh CL (2024) Practicalities of community-led continuous water quality monitoring: Lessons from Taiwan and UK pilots. Front Environ Sci 12. 10.3389/fenvs.2024.1371048.

[CR82] Taylor J, Graham M, Louw A, Lepheana A, Madikizela B, Dickens C, Chapman DV, Warner S (2022) Social change innovations, citizen science, miniSASS and the SDGs. Water Policy 24(5):708–717. 10.2166/wp.2021.264.

[CR83] Thornhill I, Chautard A, Loiselle S (2018) Monitoring biological and chemical trends in temperate stillwaters using citizen science. Water (Switzerland), 10(7). 10.3390/w10070839.

[CR85] Tumwesigye Z, Tumwesigye W, Opio F, Kemigabo C, Mujuni B (2022) The Effect of Water Quality on Aquaculture Productivity in Ibanda District, Uganda. Aquac J 2(1):23–36. 10.3390/aquacj2010003.

[CR87] Wadoux AMJ-C, McBratney AB (2023) Participatory approaches for soil research and management: A literature-based synthesis. Soil Security 10:100085. 10.1016/j.soisec.2023.100085.

[CR88] Wang S, Matt M, Murphy BL, Perkins M, Matthews DA, Moran SD, Zeng T (2020) Organic micropollutants in New York lakes: A statewide citizen science occurrence study. Environ Sci Technol 54(21):13759–13770. 10.1021/acs.est.0c04775. 33064942 10.1021/acs.est.0c04775

[CR89] Wanja DW, Mbuthia PG, Waruiru RM, Mwadime JM, Bebora LC, Nyaga PN, Ngowi HA (2020) Fish husbandry practices and water quality in Central Kenya: Potential risk factors for fish mortality and infectious diseases. Vet Med Int 2020:1–10. 10.1155/2020/6839354. 10.1155/2020/6839354 PMC7106927 32257096

[CR90] Whitehead PG, Edmunds P, Bussi G, O’Donnell S, Futter M, Groom S, Rampley C, Szweda C, Johnson D, Triggs Hodge A, Porter T, Castro G (2024) Real-time water quality forecasting in rivers using satellite data and dynamic models: An online system for operational management, control and citizen science. Front Environ Sci 12. 10.3389/fenvs.2024.1331783.

[CR91] Yardi KD, Bharucha E, Girade S (2019) Post-restoration monitoring of water quality and avifaunal diversity of Pashan Lake, Pune, India using a citizen science approach. Freshw Sci 38(2):332–341. 10.1086/703440.

[CR92] Yevenes MA, Pereira H, Bermudez R (2022) Citizen science as a co-creative measure to water quality: Chemical data and local participation in a rural territory. Front Environ Sci 10. 10.3389/fenvs.2022.940778.

